# Mapping socio-environmentally vulnerable hotspots in the Volta Delta of Ghana

**DOI:** 10.1371/journal.pone.0322453

**Published:** 2025-05-21

**Authors:** Fiifi Amoako Johnson, Philip-Neri Jayson-Quashigah, Duncan Hornby, Chris Hill, Mumuni Abu, Kwasi Appeaning Addo, Benjamin Kofi Nyarko, Samuel Nii Ardey Codjoe, Cynthia Addoquaye Tagoe, Craig William Hutton, Sabu Padmadas

**Affiliations:** 1 Department of Population and Health, University of Cape Coast, Cape Coast, Ghana; 2 Department of Marine and Fisheries Sciences, University of Ghana, Accra, Ghana; 3 GeoData Institute, University of Southampton, Southampton, United Kingdom; 4 Regional Institute for Population Studies, University of Ghana, Accra, Ghana; 5 Department of Geography and Regional Planning, University of Cape Coast, Cape Coast, Ghana; 6 Institute of Statistical, Social and Economic Research, University of Ghana, Accra, Ghana; 7 Department of Social Statistics and Demography, University of Southampton, Southampton, United Kingdom; Brunel University London, UNITED KINGDOM OF GREAT BRITAIN AND NORTHERN IRELAND

## Abstract

**Background:**

Many Delta residents are dependent on climate-sensitive resources for their survival. Nonetheless, these resources are susceptible to climatic change and variability. The Volta delta of Ghana is severely impacted by sea-level rise resulting in flooding, salinisation and permanent loss of lands, with relentless social and economic consequences. However, vulnerability assessments in the Volta Delta have primarily focused on sea level rise, with limited attention to communities’ susceptibility to adverse socio-environmental impacts. This study maps socio-environmentally vulnerable hotspots in the Volta Delta, employing methods incorporating residents, stakeholders and experts’ opinions.

**Methods:**

Vulnerability is conceptualised based on the Intergovernmental Panel on Climate Change (IPCC) climate risk and socioeconomic vulnerability framework. The framework defines vulnerability as a function of sensitivity and adaptive capacity. Through stakeholder engagements, community support systems sensitive to climate-driven stressors, referred to as sensitivity dimensions, were identified. Those that enhance local communities’ ability to adjust and mitigate the impacts of climate stressors, termed adaptive capacity dimensions, were also identified. Indicators for quantifying the dimensions were also identified through stakeholder engagements. Data for the analysis were extracted from multiple sources including Census, Landsat imagery, national land surveillance and Google Earth. Geospatial statistical techniques were used to analyse and map socio-environmental vulnerability hotspots.

**Results:**

The findings show that vulnerable communities were predominantly agrarian communities clustered along the intersections of the South Tongu, North Tongu, and Akatsi districts as well as the Dangbe West and North Tongu districts. Communities along the eastern coastline of Keta and Ketu South Municipalities and the Dangbe West District were the least vulnerable. The results also show, that although communities along the coastal lines of the Keta, Ada East and Ada West districts were highly vulnerable to sea-level rise, access to vibrant cross-country economic and industrial activities at the Ghana-Togo border and the capital city of Accra and the port city of Tema contribute to their low socio-environmental vulnerability.

**Conclusion:**

Socioeconomic ability, particularly access to alternative economic activities has the potential to mitigate vulnerability to environmental stressors. The findings direct the need for area-specific targeted and concerted interventions for strengthening the socioeconomic ability and adaptation capacity of the Volta Delta residents.

## Introduction

Vulnerability assessments are conducted globally to understand the level of risk of places prone to various stressors and hazards [1,2]. The observed increase in losses and damages resulting from the impact of hazards has shifted research attention to actions aimed at minimising the effects on vulnerable populations living in potentially hazardous environments [3–5]. It has, therefore, become necessary to examine the capabilities of human populations to reduce the impacts of hazards and enhance the socioeconomic ability of communities.

Socio-environmental vulnerability is a multidimensional concept used to identify and characterise the factors which enable communities to respond to the impacts of hazards [4,5]. Disaster losses and damages are measured not only by the magnitude and duration of the event but also by the adaptive capacity of the population to protect themselves, their livelihoods, and assets [4] and the sensitivity and exposure of the population to the immediate impacts of the given hazard [6]. In other words, socio-environmental vulnerability is dependent on the risk of exposure, the nature and magnitude of the impact on exposed systems and human society [7,8]. Thus, socio-environmental vulnerability assessment should be area-specific and systems-targeted, incorporating relevant information based on past experiences, future risks and expectations of climatic conditions and socio-economic dynamics of the exposed population [9–12]. Consequently, socio-environmental and socioeconomic indicators are critical for assessing communities’ vulnerability to hazards, but they differ across different social and spatial contexts [4,5,12–14].

Globally, coastal environments are dynamic and complex and these continue to change due to the influence of anthropogenic factors [15]. Low-lying deltas, whilst presenting diverse economic opportunities for residents, are highly prone to climate and environmental stressors [16]. Deltas are disposed to climatic hazards such as sea-level rise, erosion, high tides, storms and salinity, which when considered in the light of the increasing population in these regions and the diverse ecosystem services-based socioeconomic activities, exacerbate both the vulnerability and exposure of coastal populations to hazards [17–20].

The degree of vulnerability is not only dependent on proximity to a given hazard or the environmental characteristics of a place but also on the socioeconomic characteristics of the population in question [19–22]. High rates of population growth and urbanisation are critical issues leading to the development of informal settlements and the development of housing units in exposed environments. Research evidence shows that urban informal settlements are often neglected areas which are highly vulnerable to the impacts of climate stressors, and also congregated by populations of higher exposure and lower adaptive capacity [23–27]. Informal urban settlers are not only exposed to the direct impacts of climate variability and change but also the indirect effects including food insecurity and malnutrition, transmission of infectious diseases, depression and domestic violence, among others [28]. Evidence shows that urban development plans in many low- and middle-income countries lack comprehensive climate adaptation measures, with limited focus on the poor and informal settlements [27]. The widening disparities in wealth and socioeconomic status may bring about increasing losses and damages to disasters in the future because of the inability of the people to cope with the situation, particularly trapped and poverty-stricken communities exposed to cycles of hazards [29,30]. Generally, potential exposure to hazards spatially interacts with the existing socio-demographic characteristics to induce socio-environmental vulnerability [7,21,31].

The Volta delta of Ghana is severely impacted by sea level rise resulting in flooding, salinisation and permanent loss of lands, with relentless social and economic consequences [32,33]. However, vulnerability assessment in the Volta Delta has primarily focused on physical exposure to sea level rise [33]. There has been limited attention to communities’ susceptibility to the adverse socioeconomic impacts of climate stressors and their ability to cope, resist, and recover from these effects (socio-environmental vulnerability). This study, thus, identifies delta-specific indicators and analyses and maps climate-related socio-environmentally vulnerable hotspots in the Volta delta estuary. Although there are studies on the biophysical vulnerability of deltas [4,34–38], assessment of socio-environmental vulnerability at a higher resolution (community level) is critical for identifying distinctive deltaic populations needing help to build resilience towards environmental stressors.

The study uses relevant statistical techniques, incorporating local knowledge of the delta’s residents, stakeholders and experts to identify socio-environmentally vulnerable hotspots in the Volta Delta of Ghana. The study hypothesis that in climate-stressed deltas, access to human resources, economic alternatives and security and an enabling environment reduces the impact of climate stressors, whilst, the lack of it worsens the impacts. To explore the multi-dimensionality of socio-environmental vulnerability, the study identifies community support systems (sensitivity dimensions) susceptible to the impacts of climate and environmental stressors and the area-specific relevant indicators for quantifying their impacts on local communities. In addition, community support systems which enhance local communities’ ability to adjust and mitigate potential adverse effects, take advantage of opportunities and cope with the consequences (adaptive capacity dimensions) of climate and environmental stressors the relevant indicators for quantifying them were explored through stakeholder engagement. Given the growing and worsening impacts of climate variability and change in delta regions, a study of this nature is critical for categorising locally relevant indicators for assessing socio-environmental vulnerability and identifying distinctive populations requiring adaptation support.

### Conceptualisation of vulnerability

Local-level quantification of vulnerability remains challenging due to several factors. The determinants (biophysical, social, economic and institutional) of local level vulnerability are spatiotemporal and interact complexly [39,40]. In many low- and middle-income countries, relevant disaggregated data at the local level to quantify vulnerability are often not available [40,41]. Also, vulnerability is location-specific and highly dependent on sociocultural and economic conditions [42]. Further, although governance structures shape vulnerability at the local level, accessing reliable data on policy enforcement and government inefficiencies is often elusive and thus hard to quantify [39,42,43]. Consequently, globalised frameworks often fail to adequately capture local level vulnerability and complicate the development of standardised measures [39].

Although the Intergovernmental Panel on Climate Change (IPCC) framework may not be completely immune from these challenges, it provides a comprehensive conceptualisation that ensures a holistic understanding of how local communities are affected by climate and environmental stressors [44]. Indigenous and local knowledge has the potential to shape understanding of communities’ vulnerability to climate and environmental stressors, providing accurate and useful information for adaptation options [45–47]. The IPCC framework lends itself to the integration of scientific data, methodologies and stakeholder knowledge in quantifying local level vulnerability. Further, the dimensions of the IPCC framework facilitate the identification of adaptation strategies tailored to individual communities and offer insights relevant to policy decisions at the local level [43].

Given the above, we adopted the IPCC [48] working definition of vulnerability, which provides a practical realistic assessment and quantification of vulnerability at the local level. The IPCC [48] conceptualisation of vulnerability acknowledges the complex interactions amongst the climate, and the natural environment and their resultant impacts on human processes and wellbeing. Vulnerability is the degree or the predisposition of a system to be adversely affected by climate change due to exposure, sensitivity or susceptibility to harm and lack of capacity to cope and adapt [23,48]. Therefore, it is a function of the character, magnitude and nature of environmental hazard a system is exposed to, its sensitivity and adaptive capacity [48,49], expressed as:


$$\begin{array}{c} \text{Vulnerability }=f\left(\text{Exposure},\text{Sensitivity},\text{Adaptive capacity}\right) \end{array}$$ (1)

Exposure accounts for the presence and distribution of community systems (livelihoods, ecosystems, services and resources, infrastructure, economic, social and cultural assets) as well as human populations that could be adversely affected. Sensitivity refers to the degree to which a system could be affected by climate-related stimuli or the factors affecting a system. Adaptive capacity, on the other hand, is the ability of a system to adjust to climate-related stimuli, moderate potential damages, take advantage of opportunities or cope with the consequences.

Whist, sensitivity and adaptive capacity are intrinsic properties of a system, exposure is determined by the spatial and temporal distribution of hazards and the populations at risk [50,51]. In this case, exposure is not just a modifier of vulnerability but a primary determinant of risk. Risk is not exclusively determined by climate and weather events but also by the extent of exposure and level of vulnerability [50]. Exposure control and vulnerability reduction require distinct strategic approaches [50].

The IPCC’s vulnerability framework could be applied using qualitative and quantitative approaches [52–54]. However, each approach has its advantages and disadvantages. Qualitative methods provide a contextual understanding of local level vulnerability by exploring insights into the constructs of the framework [52]. It also facilitates the exploration of local perspectives, lived experiences, and emerging issues, thus aiding in designing socially acceptable and practically feasible interventions [52,54,55]. However, qualitative findings are subjective and lack generalizability, replicability and geographical comparability [54,56]. On the other hand, quantitative approaches are objective and replicable [57–59]. They allow for the standardisation of data and spatiotemporal comparability. They also provide metrics that could be used for local level planning, validation and monitoring of adaptation policies and strategies [60,61]. However, they do not provide conceptual understanding, are sated with data challenges, particularly at the local level and often lack local perspectives [57–59]. Given the objectives of the proposed study and to ensure replicability, geographical comparability and to support policy and decision-making at the local level, a quantitative approach, incorporating stakeholder perspectives was adopted.

Several formulations of Equation (1) are proposed in the literature [23,62–64], however, in a broader context, the definitions are similar. Despite the range of possible formulations, the IPCC’s [48] definition provides a viable working characterisation, which in practice, is flexible to implement locally, even in data-poor regions. Exposure in effect, serves mainly to scale the variability of vulnerability, spatially and temporally [49]. In this regard, the study adopted the spatially explicit vulnerability concept where the assessment of vulnerability to climate stressors considers area-specific characteristics [65]. The concept postulates that climate change impacts and adaptive capacities are not uniform across locations, as they are influenced by factors such as land use, socio-economic conditions and ecological systems, among others. Thus, for any one particular place and time, the relationship could be simplified as:


$$\begin{array}{c} \text{Vulnerability}=f\left(\text{Sensitivity, Adaptive capacity}\right) \end{array}$$ (2)

The present study adopted Equation (2) as the formulation of vulnerability for the Volta Delta. Excluding exposure in Equation (2) predisposes that differences in vulnerability are driven by variations in exposure rather than sensitivity or adaptive capacity. Thus, if exposure varies significantly across a study area, then comparison of local level vulnerability may become misleading. Nonetheless, given that the study covers a geographic area that is generally affected by sea-level rise, droughts and floods with all local communities in a similar hazard zone with minimal variations, differences in vulnerability are primarily driven by differences in sensitivity and adaptive capacity rather than exposure [66]. Holding exposure constant allows us to isolate the effects of sensitivity (how much a system is affected) and adaptive capacity (ability to cope and recover). From this perspective, the IPCC in their “Summary for policymakers” synthesis reports explicitly defined vulnerability as a function of sensitivity and adaptive capacity [67–69]. The IPCC revised vulnerability concept, defines risk as a function of hazard, exposure and vulnerability, where vulnerability is a function of sensitivity and adaptive capacity [69]. Thus, inherent community characteristics predispose local populations to the effects of environmental stressors, shaped by factors such as socio-economic conditions, cultural norms, health status, access to resources, and governance structures [5, 39]. Adopting this definition, policy and programme interventions could be tailored towards modifiable factors. Understanding internal vulnerability is crucial for effective risk management and resilience-building efforts.

In this form, vulnerability is driven mainly by socioeconomic and environmental factors. On this assumption, equation (2) is appropriate for integrating the social and environmental aspects of vulnerability. This conforms to the IPCC’s [48] narrative that socioeconomic factors are key drivers of the vulnerability and adaptability of human systems to climate change.

For targeted policy decisions, programmes and interventions, it is important to understand the impacts of risk associated with climate change by examining the combined effects of hazards, the level of socioeconomic vulnerability and the exposure of people, ecosystems and assets. Indeed, due to the multidimensionality (livelihoods, housing and ecosystem services, amongst others) of vulnerability to climate stressors, it is also imperative to evaluate the factors that regulate each component. Similarly, for quantification of sensitivity and adaptive capacity, it is important to acknowledge their multidimensionality. This is because multiple factors act collectively to contribute to the extent to which communities may be sensitive or adaptive to climate-related stimuli. Opportunities and access to resources (livelihoods, access to land, water and sanitation, healthcare, amongst others) and exposure to climate hazards (sea erosion, drought, floods, etc.) are not spatially evenly distributed [70]. Some local communities may be vulnerable because they are dependent on climate-sensitive livelihoods, for others, their water sources may be exposed, they may lose essential ecosystems or may not be able to access essential services. Similarly, some communities may be able to cope or adapt more than others because they have access to economic alternatives or high human capital. A combination of factors determines a community’s level of vulnerability. Therefore, identifying these differences is important for mitigating the impacts of climate-related hazards and stressors. In this study, we profile the different components of sensitivity and adaptive capacity, referred to hereafter as dimensions. The IPCC [48] identifies sensitivity and adaptive capacity as critical dimensions of vulnerability to climate change. These dimensions are used to assess how systems, populations, or environments respond to climate change impacts and how well they can adapt.

## Study site

Studies in coastal areas and delta systems often face the challenge of effective demarcation and resolution of study areas. Multidisciplinary perspectives considering geologic, floodplain, geopolitical and historical assessments have been used to characterise the Mississippi Delta [71]. Other studies followed a more geopolitical and physiographic perspective focusing on political and administrative boundaries to describe deltas including the Greater Pearl Delta in China, Hong Kong and Macao [72]. The Rhine-Meuse in the Netherlands, the Mekong in Vietnam, and the Ganges-Brahmaputra in Bangladesh amongst other deltas [73]. This study, based on the DECCMA project [37] definition, focuses on communities where either portion of the district or the whole district is within the land below a five-metre contour in the lower portion of the Volta River basin. Defining the delta to include land below the five-metre contour also allows the study to focus on the coastal processes and hazards linked to present conditions and the relative rise of the sea level [37].

The Volta Delta is located within the Keta basin and traverses two administrative regions (Greater Accra and Volta) of Ghana with unique socio-demographic and biophysical characteristics. The delta has diverse ethnic and cultural groups engaged in various livelihood activities [74], and they are highly exposed to sea level rise, high tides, sea erosion, salinity and drought [37].

The Volta Delta is a 400,000 kilometres square trans-national watershed in six countries (Ghana, Burkina Faso, Togo, Mali, Benin and Cote d’Ivoire). The Volta basin in Ghana constitutes 40 per cent of the river’s catchment [75,76]. The Volta Delta is located within the lower portion of the Volta River in the Accra-Ho-Keta Plains, within latitudes 5^0^25’ and 6^0^20’ North and longitude 0^0^40’ and 1^0^10’ East along the eastern coast of Ghana and covers a total area of about 4,562-kilometre square [37]. The east of the Volta Delta borders Lomé, the national capital of Togo, whilst the west shares an administrative boundary with the national capital Accra which is about 40 kilometres from the Delta and Tema, the industrial hub of Ghana located about 10 kilometres from the Delta [37].

This study covers 771 (communities) Census Enumeration Areas (EAs) within 13 administrative districts (Ada East, Ada West, Shai Osu Doku and Ningo Prampram in the Greater Accra Region and Ketu North, Ketu South, Anloga, South Tongu, North Tongu, Central Tongu, Akatsi South, Akatsi North and Keta Municipal in the Volta Region) classified according to the 2012 administrative district demarcation of Ghana.

Geographically, Ghana is demarcated into 16 regions (10 regions in 2012), each headed by a regional minister appointed by the President. At the sub-regional level, the regions are further demarcated into districts. Populous and more developed districts are generally referred to as municipalities and metropolitan areas [77]. Districts are further classified into sub-district, urban, town, area councils and unit committees [77,78]. The district administrations hold legislative power at the local level and are responsible for revenue collection, resource allocation, and planning and evaluation of development activities [78]. EAs are the smallest geographical statistical units created for Census enumeration. An EA can be a city or town block, a village, part of a village or a group of small villages or a unit committee area with well-defined boundaries identified on a map. For the 2010 Ghana Population and Housing Census (GPHC), the country was demarcated into 37,642 EAs [79]. The present analysis is conducted at the EA level.

## Data and methods

### Data

A multidimensional matrix of indicators was selected to analyse the dimensions. The indicators were selected based on existing literature, data availability, and what residents reported as appropriate, relevant and robust for each dimension. The indicators selected for each dimension and the sources of data are shown in Tables 1 and 2. The data were collated from different sources including the 2010 GPHC, Landsat data imagery, Google Earth and government sources amongst others (Table 3).

**Table 1:** Sensitivity dimensions, their selected indicators and sources of data.

Dimensions	Indicators	Source of data	Year
Livelihood			
	Percentage of the working population engaged in crop farming	Census	2010
	Percentage of the working population engaged in tree growing	Census	2010
	Percentage of the working population engaged in fish farming	Census	2010
	Percentage of the working population engaged in salt mining	Census	
	Cultivated land per capita	LULC	
Housing			
	Quality of material for the construction of the wall	Census	2010
	Quality of material for roofing	Census	2010
	Quality of material for floor	Census	2010
Health			
	The proportion of children under 5 years of age who did not survive to their fifth birthday	Census	2010
	Proportion of deaths due to pregnancy amongst women of reproductive age	Census	2010
	Percentage of the population with disabilities	Census	2010
	Malaria incidence rate	Bhatt et al, 2015 [87]	2000-2015
	Malaria parasite rate	Bhatt et al, 2015 [87]	2000-2015
	Distance to nearest health facility	Amoako Johnson et al. 2015 [86]	2000-2015
Ecosystems services			
	Natural beach area per capita	LULC	2015
	Mangrove area per capita	LULC	2015
	Riverine vegetation per capita	LULC	2015
	Muddy area per capita	LULC	2015
	Savanah grassland per capita	LULC	2015
	Rivers and streams area per capita	LULC	2015
	Reservoirs and dams area per capita	LULC	2015
	Lagoon area per capita	LULC	2015
	Salt-pans area per capita	LULC	2015
	Tidal pool area per capita	LULC	2015
	Wetlands per capita	LULC	2015
	Percentage of households dependent on wood as the main source of fuel for cooking	Census	2010
Water and sanitation			
	The main source of water for drinking	Census	2010
	The main source of water for domestic use	Census	2010
	Type of toilet facility	Census	2010
	The main method of refuse disposal	Census	2010
	The main method of liquid waste disposal	Census	2010

**Table 2:** Adaptive capacity dimensions, their selected indicators and sources of data.

Dimensions	Indicators	Source of data	Year
Access to economic alternatives and services			
	Distance to the nearest urban centre	LULC	2015
	Feeder road density per kilometre square	CERSGIS & GRHA	2013
	Trunk road density per kilometre square	CERSGIS & GRHA	2013
	Unengineered road density per kilometre square	CERSGIS & GRHA	2013
	Percentage of the working population engaged in non-agricultural activities	Census	2010
Human resource capacity			
	Percentage of population with secondary or higher education	Census	2010
	Adult literacy rate	Census	2010
	Working age group who are economically active/in school	Census	2010
	Labour market support ratio - the ratio of the population aged 15 years or older who are working to those who are not working population	Census	2010
	Percentage of the working population who are managers, professionals, technicians and associate professionals	Census	2010
Economic security/assets			
	Percentage who owns a mobile phone	Census	2010
	Percentage of households with fixed phone	Census	2010
	Percentage of households that own a desktop	Census	2010
Enabling environment			
	Proximity to coastline	Google Earth	2016
	Length of revetment	Google Earth	2016
	Proportion of coastline length covered with groynes	Google Earth	2016
	Distance to nearest freshwater body	Google Earth	2016
	Mean elevation	Google Earth	2016

Census – 2010 Ghana Population and Housing Census; CERSGIS – Centre for Remote Sensing and Geographic Information Services; GRHA – Ghana Roads and Highways Authority; LULC – Land Use and Land Classification

**Table 3:** Land Cover Classification System (LCCS) classes and the ecosystem services they provide in the Volta Delta.

LCCS classes	Ecosystem services	Source of information	Source of data	Year
Natural Beach	Sand mining and beach seine fishing (Ketu)	[109,110]	LULC	2015
Mangrove (rhizophora and avicennia mangroves)	Non-timber forest products; fish smoking; fuel wood/charcoal production; roofing materials; fishery of black tilapia; alcohol ‘akpeteshi’ distilling; harvesting crabs and molluscs and medicinal plant products	[80,83,85]	LULC	2015
Riverine vegetation	Fuel wood	[111]	LULC	2015
Muddy area	Brick-making	[112]	LULC	2015
Savanna grassland	Subsistence agriculture (cassava) and rotational bush fallow; livestock grazing; hunting and fuelwood	[104,113,114]	LULC	2015
Rivers & Streams	Freshwater fisheries, flood plain fisheries (flood ponds)	[104]	LULC	2015
Reservoirs and dams	Freshwater and energy	[104,115]	LULC	2015
Lagoon	Artisanal fishing; weaving of raffia matts (northern freshwater parts of Keta district – ketsi grass (Ketu) and shrimp and hunting	[104,116]	LULC	2015
Salt-pans/ salt flats	Salt mining/production of ‘soli’ herb for fish smoking on the margins of the salt flats	[104,114,117]	LULC	2015
Tidal pools	Artisanal fishing	[114]	LULC	2015
Wetlands	Oyster shell mining (Dangbe West/North Tongu)	[114]	LULC	2015

The 2010 GPHC is the fifth census conducted in Ghana since the country attained independence in 1957. The Census Night for the 2010 GPHC was 26th September 2010. The Census enumerated 24,658,823 people, consisting of 12,024,845 males and 12,633,978 females [79]. For the Volta delta, the Census enumerated 888,180 people, representing 3.6% of the total population of Ghana from 211,075 households. The 2010 GPHC collected data on the demographics and socioeconomic status of the population. The indicators derived from the Census data are shown in Tables 1 and 2.

Another key data source for the study is the Landsat imagery data used for deriving Land Use and Land Classification (LULC). Two scenes (193,056 and 192,056) of Landsat imagery for the year 2015 acquired from the United States Geological Survey were augmented with the United Nations Food and Agriculture Organisation’s (FAO) Global Land Cover. Using FAO classification approach, the imageries were segmented and then interpreted using the FAO Land Cover Classification System tool. The results were validated using 80 validation plots across the study area. A comprehensive accuracy assessment of the Landsat imagery-based was conducted using photo interpretation of Google Earth imagery from the same period of analysis. The Kappa statistics show an overall accuracy of 90.21% of correctly classified classes. Eighteen classes were identified for the study. The LULC was used as a proxy to assess communities’ dependence on production ecosystem services which are sources of additional or alternative livelihoods in the Volta delta, the loss of which could also be detrimental to the population. The value of ecosystem services across the delta has been estimated as US$ 340 per hectare per year ($/ha/yr) return from harvesting (including shellfish, salt, wood, medicine, and fodder, among others) from within the mangroves and a $165/ha/yr contribution to marine fisheries [80]. A number of other studies highlight alternate livelihoods from mangroves in the Volta delta [81–83]. These services are subject to loss in instances where mangrove areas are seasonally cleared and changed. UNEP [84] for example, reported a 25 percent loss in mangrove areas from 1980–2006, with fragmentation affecting the wetlands within the savannah grasslands [85]. The LULC indicators derived for the survey are shown in Tables 1 and 2, whilst Table 3 shows the Land Cover Classification System (LCCS) classes and the production ecosystem services they provide in the Volta Delta.

We used a digitised topographic database of national road networks, last updated in 2013, from a national programme of land surveillance conducted by the Centre for Remote Sensing and Geographic Information Services (CERSGIS) of the University of Ghana in collaboration with the Ghana Roads and Highways Authority (GRHA). The network includes trunk, feeder and unengineered roads. In addition, we used a georeferenced list of health facilities compiled by the Centre for Remote Sensing and Geographic Information Services (CERSGIS), University of Ghana and Amoako Johnson et al. [86] to compute the road network distance from the centroid of a community to the nearest health facility.

To examine the health impacts of environmental-related stressors in the delta, Bayesian geospatial model-based estimates of mean clinical Plasmodium falciparum malaria cases per person per annum for the year 2000–2015 and population-weighted Plasmodium falciparum parasite rate standardised to the population aged 2–10 years [87] were used to derive malaria incidence and parasite rates, respectively, for each community in the study area (Table 1). Further, the DECCMA study team used Google Earth to collate information on physically engineered adaptation and coastal land use in the Volta Delta coastline. This information was used to extract enabling environment indicators including proximity to the coastline, length of revetment within a community and the proportion of coastline length within a community that were covered with groynes.

## Methods

The dimensions were classified through a literature review, field observations, and discussions with residents, stakeholders and experts to avoid bias. The criteria for classification of the dimensions were based on what residents, stakeholders and experts deemed appropriate, relevant and robust for specific dimensions. Factor analysis, employing the maximum likelihood estimation approach was used to derive a single factor score (first factor score) for each dimension from the multi-dimensional matrix of indicators selected to represent each dimension. Factor analysis is a statistical technique that reduces many variables by extracting their commonalities into smaller factors [88]. The technique was adopted because it circumvents multicollinearity [49,89]. Before performing factor analysis, the variables were standardised by subtracting the mean from each of the actual observations in a dimension and dividing by the standard deviation. In this case, each standardised variable had a mean of 0 and a standard deviation of 1. The factor score generated through MLF analysis to represent each dimension is a unitless score [90,91]. Therefore, to ensure comparability across the dimensions, they are rescaled to values between 0 and 1 [91]. Thus, if *N* is the number of communities in the delta, the factor scores were ranked from the lowest to the highest factor, such that the ranked score $R=\frac{1}{N},\;\frac{2}{N},\frac{3}{N},\;\ldots ,\;\frac{N}{N}$ . In this regard, for the sensitivity dimensions, low scores (rankings) reflect low sensitivity, whilst high scores reflect high sensitivity. Likewise, for the adaptive capacity dimensions, low scores (rankings) reflect low adaptive capacity, whilst high scores reflect high adaptive capacity.

The statistical distribution of vulnerability scores is dependent on the characteristics of the population being studied, the indicators used and the study context. Studies have observed that vulnerability scores are usually normal, log-normal or exponentially distributed [5,39,92,93]. In many low- and middle-income countries, vulnerability scores tend to be exponentially distributed, i.e., most populations or systems exhibit low or moderate vulnerability, while a small proportion experiences high vulnerability due to compounded risk factors such as poverty, poor health, lack of access to resources and high environmental risks [49,94]. In this study, we aim to use the scores to identify levels of vulnerability, ensuring that they reflect the distribution of vulnerability within populations and they are comparable across scales. For a robust representation of communities’ level of vulnerability, the distributional properties of vulnerability were taken into account. Research evidence suggests that socio-environmental vulnerability within a population is not linearly distributed but exponentially [49,94,95]. This study examined socio-environmental vulnerability concentration within geographic areas, thus the use of an exponential distribution. The exponential distribution shows high concentration of low vulnerability scores but a long tail of higher scores indicating fewer but more extreme cases of vulnerability across population groups [92,93]. In this case, many communities may have low vulnerability scores, indicating moderate resilience, while a small proportion experience high vulnerability due to compounded risk factors such as poor health, lack of access to resources and high environmental risks. Cutter et al. (2003) [5] provide a comprehensive framework for assessing social vulnerability to hazards, highlighting how social vulnerability is distributed across populations and often clustered in smaller, disadvantaged subpopulations.

In this regard, the dimension scores were exponentially transformed, applying a scaling process that aligns the distributions of the scores across different dimensions, ensuring that they have comparable ranges (minimum and maximum values) for easier comparison. This helps to identify the most sensitive and the least adaptive communities. The exponential transformation procedure adopted incorporates a ‘cancellation property’, which ensures that high scores in one dimension do not cancel out low scores in others [95]. This property is highly desirable since the dimension scores are combined to identify highly vulnerable areas. When dimension scores are combined to generate a single score, a major concern is, to what extent should high scores in one dimension cancel out low scores in another dimension? For example, if a community has high livelihood sensitivity but low health sensitivity, should the latter cancel out the former and to what extent? The methodology adopted is formulated to mitigate the potential for scores in one dimension to completely nullify the scores in another dimension. This helps ensure that each dimension’s scores contribute meaningfully to the overall scores, rather than being nullified by scores from other dimensions. The exponential transformation regulates how much influence scores from one dimension can have in nullifying scores from another dimension. A deliberate adjustment was incorporated to ensure that each dimension’s contribution to the overall assessment remains balanced and meaningful, without one dimension disproportionately overshadowing others due to score cancellations.

The formulation of the exponential transformation procedure [95] adopted for this study is shown in Equation (3)


$$d_{k}=-23.026*log\left\{1-R_{i\;}*\left[1-\frac{-\lambda }{e^{23.026}}\right]\right\}\;$$ (3)

where *d*_*k*_ is the transformed dimension score which ranges between 0 and 100, −23.026 is a mathematical constant which gives a 10% cancellation property, log is the natural logarithm, *R*_*i*_ is the ranked scores, *e* is the exponential transformation function and the parameter λ=100 controls the degree of progression.

The dimension scores $d_{k}$ were combined, normalised, rank scaled and exponentially transformed to derive an overall sensitivity score and also adaptive capacity score [91,96]. The dimension-specific scores were weighted to reflect their severity and or importance. There are several prepositions in the literature on how this can be achieved – theoretical, empirical, policy-driven, consensus or purely arbitrary [63]. We followed a Delphi process to generate the dimension weights while ensuring that the weights adequately reflect the severity or importance of the dimensions as perceived by residents, stakeholders and experts. The Delphi technique is a systematic and interactive technique for obtaining individual opinions and building consensus on a particular issue [49,97]. We asked residents, stakeholders and experts to rank the dimensions. Each participant was asked to assign a total of 40 tick marks to rank the dimensions, with the most important dimension receiving the highest number of tick marks and the least important receiving the lowest number of tick marks. The mean scores assigned to each dimension were then computed and presented to the participants. Further deliberations were undertaken to ensure that at least 95% of the participants agreed with the rankings. The weightings (*w*_*k*_) for the dimensions are derived as the mean marks for each dimension, Equation (4)


$$w_{k}=t_{k}\;\;╱\;\;n\;$$ (4)

where $t_{k}$ is the total score for dimension *k* and *n* is the number of participants. Overall sensitivity (S) and adaptive capacity (AD) scores were then derived using equations (5) and (6)


$$S=-23.026*log\left\{1-R_{i}\left(\frac{1}{n_{l}}\sum {\mathrm{\backslash nolimits}}_{l=1}^{l}d_{i}w_{k}\right)*\left[1-\frac{-\lambda }{e^{23.026}}\right]\right\}$$ (5)

$$AD=-23.026*log\left\{1-R_{i}\left(\frac{1}{n_{h}}\sum {\mathrm{\backslash nolimits}}_{h=1}^{h}d_{j}w_{k}\right)*\left[1-\frac{-\lambda }{e^{23.026}}\right]\right\}$$ (6)

where $\frac{1}{n_{l}}\sum {\mathrm{\backslash nolimits}}_{l=1}^{l}d_{i}w_{k}$ and $\frac{1}{n_{h}}\sum {\mathrm{\backslash nolimits}}_{h=1}^{h}d_{j}w_{k}$ are the weighted average sensitivity and adaptive capacity scores, $n_{l}$ is the number of sensitivity dimensions and $n_{h}$ is the number of adaptive capacity dimensions [95]. The sensitivity and adaptive capacity scores are then used to derive an overall index of vulnerability (*D*). An inverse relationship is suggested between sensitivity and adaptive capacity [23], as shown in Equation (7)


$$D=-23.026*log\left\{1-R_{i}\left(\frac{S}{AD}\right)*\left[1-\frac{-\lambda }{e^{23.026}}\right]\right\}$$ (7)

**Fig 1:**
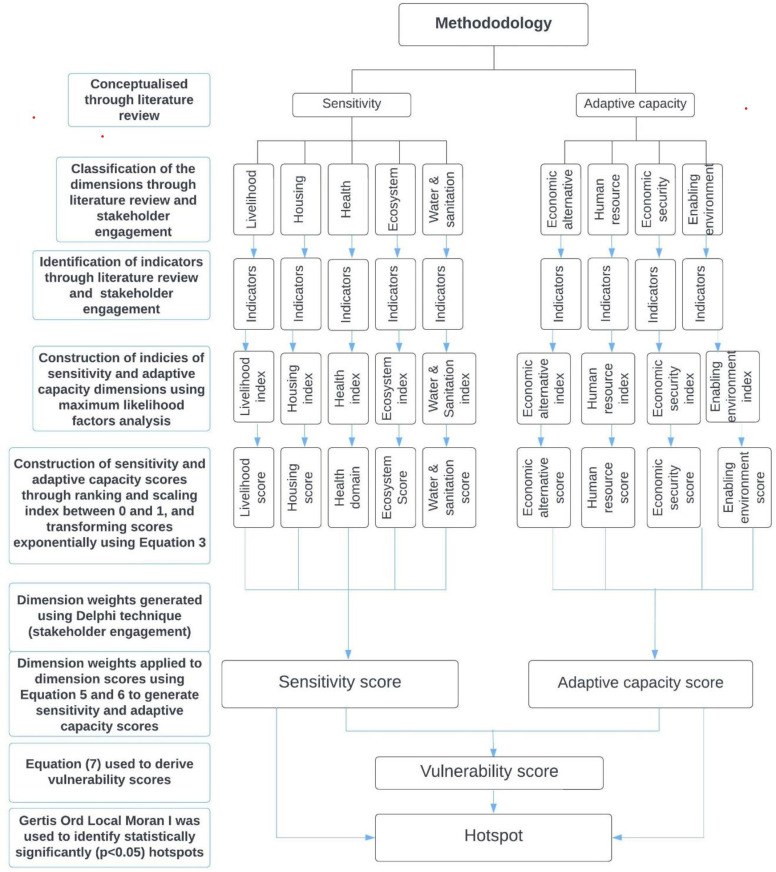
Flowchart of the analytical process adopted for the study.

Following the scores derived using equations (5), (6) and (7), the Getis Ord Local Moran I spatial autocorrelation statistical technique [98] was used to detect hotspots (spatial clustering of communities) of socio-environmental vulnerability (sensitivity, adaptative capacity and vulnerability) in the Volta delta using ArcGIS 10.7.1. The local G_i_*(d) statistic was used to identify statistically significant (p < 0.05) spatial clusters of high values (high sensitivity, adaptive capacity and vulnerability) and low values (low sensitivity, adaptive capacity and vulnerability) [98]. Where the local G_i_*(d) statistic was not statistically significant (p > 0.05), there was no spatial clustering [98]. Fig 1 shows a flowchart of the analytical process. The output maps were ground-truthed through stakeholder engagements (District Planning Officers) to elicit their views on their representativeness as well as the attributable factors.

## Results

### Sensitivity dimensions

Table 4 shows the sensitivity dimensions and their impacts as evidenced by the literature and alluded to by residents, stakeholders and experts. The sensitivity dimensions related to livelihoods, housing, health, water and sanitation and ecosystem services. Regarding livelihoods, communities in the Volta Delta with high dependence on agriculture, salt mining and fish farming were reported to be most affected by climate variability and change. Limited opportunities in non-climate dependent sectors compound the sensitivity of the delta residents. Communities with low-quality housing were reported to be less resilient to the impacts of climate threats. It was also reported that communities with unimproved water sources and poor sanitation facilities were often the most affected by climate effects. Concerning ecosystem services, populations with high dependence on ecosystem services in the Volta Delta were reported to be the most sensitive to environmental hazards.

**Table 4:** Excerpts from discussions with residents, stakeholders and experts on the sensitivity and adaptive capacity dimensions.

Dimensions	Impact and influence
*Sensitivity dimensions*	
Livelihoods	Subsistence agriculture, salt mining and fishing are the main sources of income and livelihood for most of the delta residents, however, they are highly susceptible to drought and floods including the loss of farmland, produce and equipment. Communities with high dependence on agriculture, salt mining and fish farming are often the most affected.
Housing	Climate-induced hazards such as flooding and sea erosion often destroy housing, leading to population displacements. They also affect directly and indirectly the livelihoods and health of delta populations. Communities with low-quality housing are less resilient to the impacts of climate hazards.
Health	The health impacts of climate-related hazards are both physiological and psychological. There are immediate, medium and long-term impacts on communities. The immediate impacts include injuries, deaths, and loss of health infrastructure, essential medicines and livelihoods. The medium-term impacts are infections including faecal-oral diseases, vector and rodent-borne diseases, complications of injury, anxiety and depression, communicable diseases and starvation. The long-term impacts include chronic disease, disability, mental health issues, malnutrition and poverty.
Water and sanitation	Field observations and discussions with residents and stakeholders revealed that some communities are reliant on poor water sources and drainage facilities not robust enough to stand the impacts of climate and environmental hazards. In some communities, drinking water sources submerge and or become contaminated during floods. Communities with unimproved water sources and poor sanitation facilities are often the most affected.
Dependence on ecosystem services	A large proportion of residents in the delta are dependent on ecosystem services for income, food, healthcare (medicine), fuel, and raw materials, amongst others. Extensively, these provisioning services are traded in markets and many rural households are directly dependent on them for their livelihoods. Communities with high dependence on ecosystem services are the most affected in the event of environmental hazards.
*Adaptive capacity dimensions*	
Access to economic alternatives and services	Climate and environmental stressors pose serious risks to available livelihood opportunities in the delta, particularly agriculture, salt mining and fishing and threaten any progress toward eradicating poverty. Access to alternative income-generating activities and services including health care are vital for coping with the impacts of environmental stressors. Communities close, those with access to roads, markets and services have better alternative economic opportunities.
Human resource capacity	The human resource capacity available to a community is vital for knowledge about options, accessing opportunities, advocating for support and implementing suitable and sustainable options. Education, literacy and participation in the labour market were identified as some of the key drivers of a community’s human capital to cope or adapt to the impacts of environmental stressors. Communities with high human capital are more adaptive.
Economic capacity/security	Individuals, households and communities’ economic capital are key to coping or adapting to the impacts of climate hazards. Investments in traditional sectors such as agriculture and fishing, which most of the delta’s residents depend on could have improved outputs, but they are susceptible to climatic impacts. In the Volta Delta, economic opportunities in non-climate sensitive sectors are highly limited, there is a reluctance to invest in at-risk areas, and sectors further compound the impacts of environmental stressors. Livelihood insecurity and economic poverty of marginalised communities who are most dependent on climate-sensitive livelihoods are left in a vicious cycle of poverty. Communities with high dependence on climate-sensitive livelihoods such as agriculture, fishing and salt mining are often the most affected.
Enabling environment	In communities where the risks of climate hazards are minimised and well managed the impacts are trivial. Creating enabling environments for the delta’s residence, such as building sea defences, is underpinned by Government policies, decisions, and actions. Remote communities are often not able to connect with both local and central government to benefit from programmes that create enabling environments for them.
Social capital	Household’s and communities’ ability to act collectively and timely determines the inherent capacity to mobilise resources and adapt to the impacts of climate hazards. Strong social networks enhance information sharing, boost community support, stimulate quick response and are vital for coping with environmental stressors. Communities with strong social networks and civic society groups have an intrinsic ability to cope with environmental stressors.

### Adaptive capacity dimensions

Table 4 also shows the adaptive capacity dimensions and their influence as postulated in the literature and discussed by residents, stakeholders and experts. The adaptive capacity dimensions reflected access to economic alternatives and services, human resource capacity, economic capital, enabling environment and social capital. Residents, stakeholders and experts alluded that communities near main settlements (cities), those with access to roads, markets and services have better access to alternative economic opportunities, aside from those available within the delta, thus, making them more resilient to the impact of climate stressors. Also, communities with high human resource capacity are more adaptive, and resourced with vital knowledge about options, accessing opportunities, advocating for support and implementing suitable and sustainable options. In climate-sensitive regions such as the Volta Delta, economic capital is key for adapting to the impacts of environmental change. Creating enabling environments through the provision of preventive and protective mechanisms such as sea defences were claimed to be key to coping with the impact of climate stressors. Further, it was reported that strong social networks and civic society groups within communities promote inherent abilities to cope with environmental stressors.

### Delphi ranking of the dimensions

**Fig 2:**
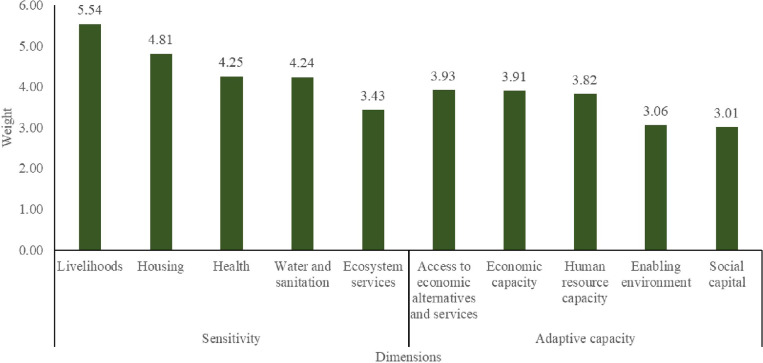
Dimension weights assigned by residents, experts and stakeholders.

The sensitivity and adaptive capacity dimensions identified were ranked by 67 residents, stakeholders and experts working in the Volta delta using the Delphi process to reflect their importance and severity (Fig 2). After the first round of the Delphi process, the mean scores and their standard deviations were presented to the residents, stakeholders and experts. Following the presentation and discussions, there was consensus that the assigned scores were reflective of the importance and severity of the dimensions. Livelihood sensitivity was ranked (mean = 5.54, standard deviation = 1.62) as the most sensitive dimension in the event of climate hazards and environmental stressors. With regards to the sensitivity dimensions, the rankings revealed that housing (mean = 4.81; standard deviation = 1.27), health (mean = 4.25, standard deviation = 1.68), water and sanitation (mean = 4.24, standard deviation = 1.28) and loss of ecosystem services (mean = 3.43, standard deviation = 1.46) were also major concerns to residents, stakeholders and experts.

Regarding the adaptive capacity dimensions, access to economic alternatives (mean = 3.93, standard deviation = 1.37), economic capacity (mean = 3.91, standard deviation = 1.31) and human resource capacity (mean = 3.82, standard deviation = 1.24) were similarly weighted reflecting their important for coping with the impact of climate threats. Further, residents, stakeholders and experts considered enabling environment (mean = 3.06, standard deviation = 1.18) and social capital (mean = 3.01, standard deviation = 1.19) as important coping mechanisms.

### Multidimensional hotspots of sensitivity and adaptive capacity

**Fig 3:**
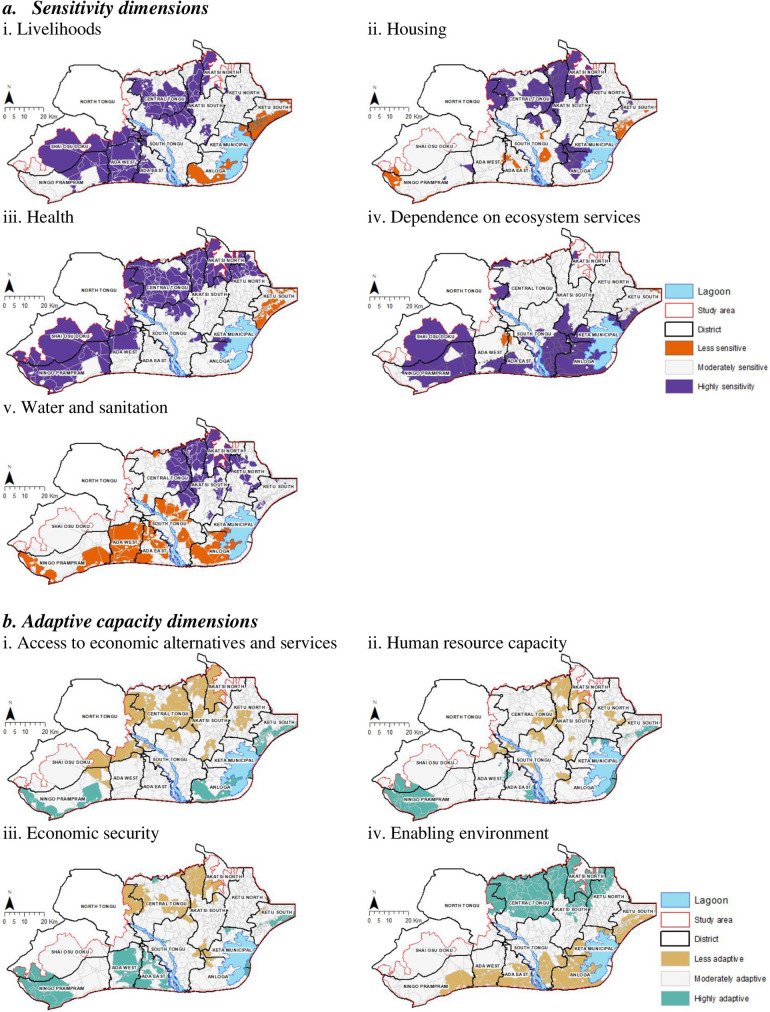
Geographical clustering of the sensitivity and adaptive capacity dimensions. a. Sensitivity dimensions. i. Livelihoods, ii. Housing, iii. Health, iv. Dependence on ecosystem services, v. Water and sanitation. b. Adaptive capacity dimensions. i. Access to economic alternatives and services, ii. Human resource capacity, iii. Economic security, iv. Enabling environment.

Fig 3 shows the geographical clustering of communities of the dimensions of sensitivity and adaptive capacity. The figure shows statistically significant (p < 0.05) clustering of communities. Those classified as highly sensitive and less adaptive were those geospatially clustered with statistically significantly high sensitivity and low adaptive capacity scores, respectively. Fig 3 shows that both sensitivity and adaptive capacity at the dimension level were not randomly distributed but clustered. The results show that high and low levels of sensitivity and adaptive capacity are not limited to particular communities in the Volta delta, but vary depending on the dimension of interest. These results are often masked when the multidimensionality of socio-environmental vulnerability to climate and environmental stressors is not considered.

Fig 3a shows the clustering of communities whose livelihoods, housing, health, ecosystems and water and sanitation infrastructure and services in the Volta delta are highly sensitive. The results show that highly sensitive with regards to livelihoods, housing, health, water and sanitation are generally clustered towards the northern and western parts of the delta. Whereas, those with high dependence on sensitive ecosystem services are generally found closer to the coast. Fig 3a further shows that the less sensitive communities are clustered around the western parts of the Shai Osu Doku and Ningo Prampram districts, and also along the coast close to the Ghana-Togo border.

Considering livelihoods, Fig 3a shows a strong clustering of communities dependent on sensitive livelihoods at the intersections of the shai Osu Doku, Prampram and Ada West and East districts. The communities with highly sensitive housing infrastructure to climate hazards and environmental stressors are clustered near the borders of North and Central Tongu, Central Tongu and Akasti North and South as well as between Keta Municipal and Anloga District. Communities sensitive to the health impacts of environmental hazards and stressors are clustered towards the northern parts of the delta, whilst those along the coast are more sensitive to the loss of ecosystem services. Communities whose water and sanitation facilities are sensitive to climate hazards and environmental stressors are observed to cluster at the intersections of Central Tongu and Akasti North and South districts as well as Ketu North district.

Fig 3b shows communities’ level of adaptability with regard to access to economic alternatives and services, human resource capacity, economic security and enabling environment. The results show that communities within the northern part of the delta are the least adaptive with regard to access to economic alternatives and services, human resource capacity and economic security (Fig 3b). On the other hand, those along the coast are the least adaptive with regard to having an enabling environment. The results show that communities near the capital city of Accra and the economically vibrant port city of Tema were the most adaptive considering access to economic alternatives and services, human resource capacity and economic security (Fig 3b).

### Sensitivity, adaptive capacity and vulnerability hotspots

**Fig 4:**
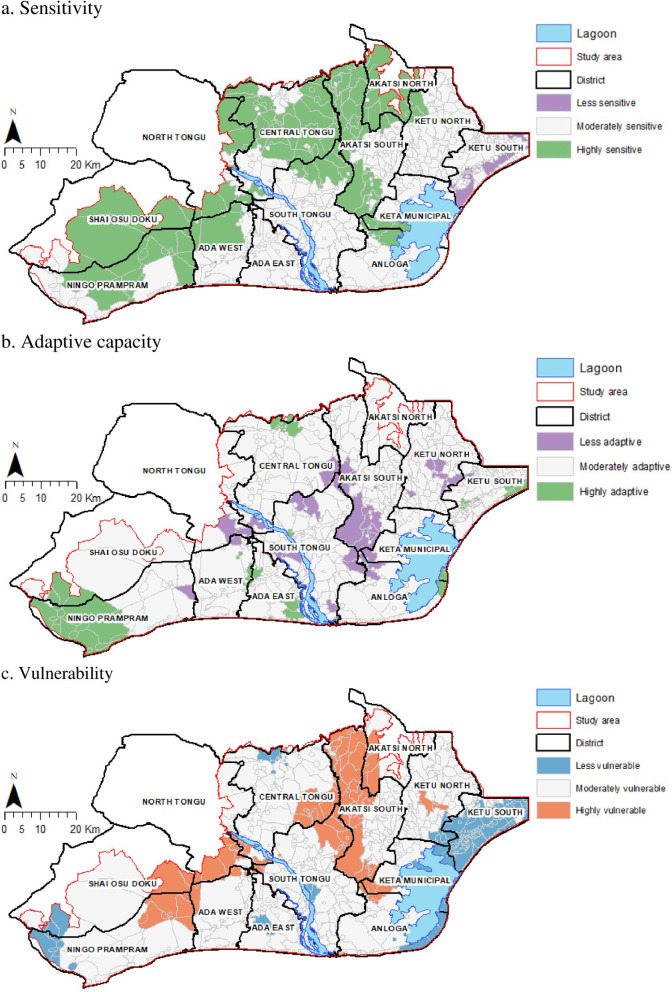
Geographical variations in communities’ level of sensitivity, adaptive capacity and vulnerability. a. Sensitivity, b. Adaptive capacity, c. Vulnerability.

Figs 4a and 4b show the geographical clustering of communities’ overall sensitivity and adaptive capacity to climate and environmental stressors based on spatial autocorrelation analysis of the weighted scores. The most sensitive communities were clustered towards the northern and western parts of the delta, particularly those in the Shai Osu Doku, Ningo Prampram, Central Tongu, Akatsi South and North districts (Fig 4a). On the other hand, communities with high adaptive capacity are clustered within the Prampram and Shai Osu Doku districts, close to the capital city of Accra and the industrial hub of Tema (Fig 4b). The least adaptive clustering of communities was identified at the intersections of the North Tongu, South Tongu and Akasti South districts.

Fig 4c shows the results from spatial autocorrelation analysis of geographical clustering of the least and the most vulnerable communities in the Volta delta. There is a strong clustering of the most vulnerable communities at the intersections of the North Tongu, Shai Osu Doku and Prampram districts, and also at the intersections of Central and South Tongu and Akasti South and North districts. The least vulnerable communities are those close to the coast and also to the Ghana-Togo border in the Ketu South Municipality and Anloga District as well as those in the Ningo Prampram and Shai Osu Doku districts, close to the capital city of Accra and the economically vibrant port city of Tema in the Greater Accra region.

## Discussions

The diverse nature, structural make-up and differential opportunities in deltas expose its residents to socio-environmental threats [99,100]. Previous studies examining socio-environmental vulnerability in deltas have overlooked the knowledge and experiences of residents and stakeholders, as well as the geographical differences crucial for designing area-specific targeted interventions. This study, drawing on the IPCC’s [48] conceptualisation of climate and social vulnerability has propositioned an integrated statistical methodology that incorporates residents, stakeholders and expert knowledge to map hotspots of the multidimensionality of socio-environmental vulnerability in the Volta delta of Ghana. The statistical methodology adopted enables appropriate comparison of scores across dimensions and also ensures that the derived scores follow the expected distribution within a population. Further, the approaches and statistical procedures prepositioned in this study could be adapted for vulnerability studies in both delta and non-delta regions.

Through engagements with residents, stakeholders and experts, the study identified that livelihoods, housing, health, ecosystem services and water and sanitation facilities are climate-sensitive community support systems susceptible (sensitivity dimensions) to climatic and environmental threats in the Volta Delta. Research evidence suggests that climate change adaptation in deltas is a complicated process where these limiters if not addressed systematically restrict adaptation processes [48,101]. The study also identified that access to economic alternatives, human resource capacity, economic capacity, enabling environment, and social capital are societal capitals that enhance local communities’ ability (adaptive capacity) to withstand and take advantage of climate and environmental stressors. These enablers are crucial for reducing or eliminating obstacles to climate change adaptation by stimulating collective abilities to adapt to the impacts of present and future climate change and variability [48,101]. Therefore, identifying these limiters (sensitivity dimensions) and enablers (adaptive capacity dimensions) through the experiences of residents, stakeholders and experts is an important research contribution to promoting adaption research and response in deltas.

Applying the integrated statistical methodology, our findings demonstrate evidence of a cluster of socio-environmentally vulnerable hotspots of communities along the intersections of the South Tongu, North Tongu and Akatsi Districts, and the Dangbe West and North Tongu districts. During the evaluation, residents, stakeholders and experts reported that the vulnerable communities in these districts are highly dependent on agriculture and the impact of persistent flooding and lack of dams continue to affect livelihoods. They further opined that lack of amenities such as portable drinking water, and access to improved roads, communication, markets, health and educational infrastructure compound their vulnerability. Additionally, social issues such as chieftaincy disputes and the practice of the Trokosi system, where virgin girls are sent to shrines to atone for crimes committed by family members [102], contribute to their social vulnerability. Stakeholders also mentioned inadequate security and destruction of crops and water by nomadic herdsmen further contribute to socio-environmental vulnerability in the area.

Further, stakeholders attributed the low vulnerability along the eastern coastline of the Keta and Ketu South districts to the vibrant cross-country economic and industrial activities at the Ghana-Togo border. Additionally, although physically vulnerable to sea-level rise [103] and flooding, booming tourist businesses in communities along the coastal lines of the Keta, Ada East and Ada West districts enhance their socioeconomic ability [104]. Similarly, high levels of economic activities in the capital city of Accra and the port city of Tema contribute to the low vulnerability identified amongst communities in the Dangbe West District. Likewise, the Dangbe West District which hosts the largest salt pan in the subregion offers alternative economic opportunities. Similar studies concur that livelihood diversification and improved physical and social capital promote resilience to climate variability and change even in communities where exposure is high [23].

Generally, stakeholders attributed the observed hotspots of socio-environmental vulnerability in the Volta delta to dependence on climate-sensitive agriculture, lack of amenities, social practices and disputes and lack of security and conflicts over resources. These observations are not unique to the Volta Delta, as deltas across the world are susceptible to sea-level rise, storm surges, saltwater intrusion and floods, coupled with anticipated increases in rainfall variability, dependence on climate-sensitive agriculture confronts its residents with challenges of food insecurity, rising food prices which further promotes poverty and inequalities [105,106]. Similarly, as reported by stakeholders in the Volta delta and also observed across many regions of sub-Saharan Africa, climate change and variability have intensified competition for resources such as land, water and fodder, among others, culminating into conflicts and social disputes compounding the socio-environmental vulnerability of delta residents [107,108]. Peculiar to socio-environmentally hotspots of the Volta delta, are the relational consequences of climate change and cultural practices (Trokosi) where dependence on climate-sensitive livelihoods, the lack of and conflict over resources increases the security risk of women and girls [102].

These findings suggest that social ability has the potential to mitigate environmental vulnerability in deltas. Our findings direct the need for area-specific targeted and concerted interventions at the local level for strengthening the social ability and adaptation capacity of Delta residents.

## Conclusions

The study proposes an integrated statistical methodology that incorporates local knowledge to map hotspots of socio-environmental vulnerability. Climate-sensitive community support systems susceptible to climatic and environmental threats identified through engagement with local stakeholders in the Volta Delta include livelihoods, housing, health, ecosystem services and water and sanitation facilities. They also reported that societal capitals that act as enablers to climate and environmental stressors in the delta includes access to economic alternatives, human resource capacity, economic capacity, enabling environment, and social capital. Applying the integrated methodology, the study observed a cluster of socio-environmentally vulnerable hotspots of communities along the intersections of the South Tongu, North Tongu and Akatsi Districts, and the Dangbe West and North Tongu districts. High dependence on climate-sensitive agriculture, lack of amenities and social challenges contributed to the observed socio-environmental vulnerability in the delta. Although highly vulnerable to sea-level rise, the study revealed that the coastal regions of Keta and Ada were less socio-environmentally vulnerable. Stakeholders attributed this to opportunities for diversification of livelihoods, access to improved physical amenities and social capital. The study concludes that enhancing social ability is a crucial adaptation to climate threats.

## Limitations

A major limitation of the study is that the proposed methodology is data-driven. Thus, its application in data-scarce regions may be limited. Concerning this, ten dimensions were identified through a review of literature and engagements with residents, stakeholders and experts, however, only nine dimensions (excluding social capital) were analysed due to data limitations. Nonetheless, the study provides a robust list of indicators where the availability of a subset could be used to examine the multidimensionality of socio-environmental vulnerability. Future studies should examine how social capital impacts the geospatial distribution of socio-environmental vulnerability hotspots and how the multidimensionality of socio-environmental vulnerability varies across regions.
